# A pantropical analysis of the impacts of forest degradation and conversion on local temperature

**DOI:** 10.1002/ece3.3262

**Published:** 2017-08-30

**Authors:** Rebecca A. Senior, Jane K. Hill, Pamela González del Pliego, Laurel K. Goode, David P. Edwards

**Affiliations:** ^1^ Department of Animal and Plant Sciences, Alfred Denny Building University of Sheffield, Western Bank Sheffield UK; ^2^ Department of Biology University of York, Wentworth Way York UK; ^3^ Department of Human Services and Oregon Health Authority Salem OR USA

**Keywords:** land‐use change, climate change, scale, tropics, temperature, thermal

## Abstract

Temperature is a core component of a species' fundamental niche. At the fine scale over which most organisms experience climate (mm to ha), temperature depends upon the amount of radiation reaching the Earth's surface, which is principally governed by vegetation. Tropical regions have undergone widespread and extreme changes to vegetation, particularly through the degradation and conversion of rainforests. As most terrestrial biodiversity is in the tropics, and many of these species possess narrow thermal limits, it is important to identify local thermal impacts of rainforest degradation and conversion. We collected pantropical, site‐level (<1 ha) temperature data from the literature to quantify impacts of land‐use change on local temperatures, and to examine whether this relationship differed aboveground relative to belowground and between wet and dry seasons. We found that local temperature in our sample sites was higher than primary forest in all human‐impacted land‐use types (N = 113,894 daytime temperature measurements from 25 studies). Warming was pronounced following conversion of forest to agricultural land (minimum +1.6°C, maximum +13.6°C), but minimal and nonsignificant when compared to forest degradation (e.g., by selective logging; minimum +1°C, maximum +1.1°C). The effect was buffered belowground (minimum buffering 0°C, maximum buffering 11.4°C), whereas seasonality had minimal impact (maximum buffering 1.9°C). We conclude that forest‐dependent species that persist following conversion of rainforest have experienced substantial local warming. Deforestation pushes these species closer to their thermal limits, making it more likely that compounding effects of future perturbations, such as severe droughts and global warming, will exceed species' tolerances. By contrast, degraded forests and belowground habitats may provide important refugia for thermally restricted species in landscapes dominated by agricultural land.

## 1 INTRODUCTION

It is well established that temperature is important in ecology, for everything from biochemistry, to physiology, to biogeography (Kearney, Shine, Porter, & Wake, 2009; Kingsolver, 2009; Puurtinen et al., 2015; Thomas et al., 2004). Temperature is a key explanatory variable in species distribution models that predict the likely impacts of projected global climate change on biodiversity (e.g., Thomas et al., 2004). However, the majority of organisms experience temperature at much finer spatial scale (Gillingham, 2010; Suggitt et al., 2011) than assumed in species distribution models (often >100 km^2^), and at local scales, temperature is more dependent on local factors (Suggitt et al., 2011) than on regional or global atmospheric circulation (Davin & De Noblet‐Ducoudr, 2010; Oke, 1987; Pielke et al., 2011; Wiens & Bachelet, 2010). One such local factor is vegetation cover, which influences temperature through direct absorption and reflection of incident solar radiation (Murcia, 1995; Oke, 1987; Snyder, Foley, Hitchman, & Delire, 2004) and through evapotranspiration, by determining the amount of thermal energy dissipated through the evaporation of water as opposed to a change in temperature (Findell, Shevliakova, Milly, & Stouffer, 2007; Lawrence & Vandecar, 2015; Oke, 1987).

Land‐use change can profoundly influence vegetation cover. Current and future land‐use change is concentrated in the tropics, where >150 million hectares of forest was converted between 1980 and 2012 (Gibbs et al., 2010; Hansen et al., 2013) and 20% of the humid tropical biome was selectively logged from 2000 to 2005 (Asner, Rudel, Aide, Defries, & Emerson, 2009). Previous studies, from a range of disciplines, demonstrate that land‐use change in the tropics tends to increase temperature (Davin & De Noblet‐Ducoudr, 2010; Findell et al., 2007; Lawrence & Vandecar, 2015; Loarie et al., 2009; Luskin & Potts, 2011; Pielke et al., 2011; Ramdani, Moffiet, & Hino, 2014). This suggests severe consequences for global terrestrial biodiversity, most of which is found in tropical rainforests (Myers, Mittermeier, Mittermeier, Da Fonseca, & Kent, 2000) and is thought to be especially sensitive to temperature change, owing to narrow thermal limits (Deutsch et al., 2008; Kingsolver, 2009; Tewksbury, Huey, & Deutsch, 2008).

Additionally, while absolute warming from global climate change will be highest at the poles (IPCC 2013), it is the tropics where relative warming will be greatest, with historically unprecedented temperatures occurring by 2050 (Mora et al., 2013). It is frequently stated that habitat fragmentation from land‐use change will make it increasingly difficult for tropical species to track climate (Brook, Sodhi, & Bradshaw, 2008; Scriven, Hodgson, Mcclean, & Hill, 2015), hampered by the poor dispersal ability of many tropical species (Van Houtan, Pimm, Halley, Bierregaard, & Lovejoy, 2007) and shallow latitudinal temperature gradients (Colwell, Brehm, Cardelús, Gilman, & Longino, 2008). However, it is less commonly discussed that the baseline temperature onto which global climate predictions are projected might itself be dramatically higher in altered land‐use types (Foster et al., 2011; Tuff, Tuff, & Davies, 2016).

To understand current and future consequences for tropical biodiversity from land‐use change and climate change, it is vital to understand thermal change at the scale at which temperature is experienced by organisms (Gillingham, 2010; Suggitt et al., 2011; Wiens & Bachelet, 2010). Prior evidence for local warming in the tropics as a result of land‐use change originates from global General Circulation Models (Davin & De Noblet‐Ducoudr, 2010; Findell et al., 2007; Pielke et al., 2011) and observational studies focused on particular locations, such as Brazil (Loarie et al., 2009), Malaysia (Luskin & Potts, 2011), and Indonesia (Ramdani et al., 2014). While General Circulation Models are limited in biological relevance by their coarse spatial resolution, observational studies are limited in generality by the site‐specificity required to achieve their fine spatial resolution (Li et al., 2015). Any studies that utilize meteorological station data have limited biological relevance because stations are specifically positioned to minimize the influence of the very same local characteristics that are important to local biota, such as vegetation cover, slope, and aspect (Frenne & Verheyen, 2016).

There are several conditions under which local warming due to land‐use change might be ameliorated, which have yet to be explicitly tested. We hypothesize that low intensity forest degradation, including commercial selective logging, fragmentation, and forest regrowth (Lewis, Edwards, & Galbraith, 2015), will correspond to relatively little net change in vegetation, and hence a smaller difference in temperature. Any warming effects of land‐use change are likely reversed at night, as habitats with relatively low vegetation cover will radiate heat back to the atmosphere more freely (Chen, Franklin, & Spies, 1995; Oke, 1987). Water availability is fundamental in determining how much thermal energy can be dissipated through evaporation, and so we also expect that warming would be less during the wet season given the high water availability (and more cloudy weather) relative to dry season, and belowground relative to aboveground. In the latter case, even when water availability is very low, soil buffers external temperature change (Scheffers, Evans, Williams, & Edwards, 2014) because soil has a higher specific heat capacity than air and thus requires a greater change in thermal energy to achieve the same change in temperature (Oke, 1987).

In this study, we carry out analyses of published data to test the effect of land‐use change on local temperature across the tropics. We collected local, in situ temperature data from the literature for paired sites (<1 ha) that differed in land‐use type. Categories of land use we studied were primary forest, degraded forest, plantation, pasture, and cropland (Table 1; modified from Extended Data Table 1 in Newbold et al., 2015). We examine how land‐use change affects daytime temperature at fine‐scale spatial resolution, and we quantify the effects of: (1) forest conversion compared with forest degradation; (2) belowground compared to aboveground; and (3) wet season conditions compared to the dry season. We focus on daytime temperatures because few studies collected nighttime temperature, although we also separately test how the latter is impacted by land‐use change for the subset of studies able to provide these data. Recent studies also highlight the importance of climatic extremes for species' survival (e.g., Christidis, Stott, Hegerl, & Betts, 2013; Deutsch et al., 2008); hence, we conduct additional analyses for those studies that provide these data.

**Table 1:** Land‐use classification definitions (modified from Extended Data Table 1 in Newbold et al., 2015)

Land‐use type	Definition
Primary forest	Forest where any disturbances identified are very minor (e.g., a trail or path) or very limited in the scope of their effect (e.g., hunting of a particular species of limited ecological importance).
Degraded forest	Forest with one or more disturbances ranging from moderate intensity/breadth of impact (e.g., selective logging and bushmeat extraction), to severe intensity/breadth of impact (e.g., regrowth after clear‐felling).
Plantation forest	Extensively managed or mixed timber, fruit/coffee, oil‐palm, or rubber plantations.
Cropland	Farming for herbaceous crops, without presence of livestock.
Pasture	Farming of livestock.

## 2 METHODS

### 2.1 Literature search

We collated temperature data from peer‐reviewed literature using ISI Web of Knowledge. The search terms were as follows: “tropic*” AND (“temperature” OR “local climate”) AND (“land use” OR landuse OR “land cover” OR landcover OR urban* OR city OR cities OR agri* OR arable OR built* OR metropol* OR deforest* OR forest*) AND (change OR expansion OR growth OR encroach* OR modif* OR conversion OR convert*). We refined the search output by including only the following research areas: “environmental sciences ecology,” “remote sensing,” “agriculture,” “biodiversity conservation,” “forestry,” “urban studies”; this returned 1,372 published studies. Excluding book chapters (21) and articles that were deemed irrelevant based on the title (298) or abstract (484) reduced the total to 525 articles. We reviewed each of these articles manually. Additional unpublished data (two studies) were also provided by co‐authors (P.G., L.K.G.).

### 2.2 Selection criteria

All data originated from studies with at least two different sites in at least two different land‐use types. Sites were located between 23.44° North and South, and the natural vegetation type was defined by authors as forest. Sites were fully contained within the land‐use type of interest and positioned beneath the canopy (where applicable). Within a single study, sampling methodology was consistent across all sites and land‐use types. Differences between studies, such as soil depth or the use of radiation shields for data loggers, were accounted for by the analytical approach (see “Statistical analysis”). All sites within a single study differed in elevation by no more than 150 m.

Data collected through remote sensing or from meteorological stations were excluded, because they are inherently unrepresentative of local climatic conditions in forested areas. Meteorological stations are established to strategically avoid the very same local conditions in which we are primarily interested (Frenne & Verheyen, 2016). Acceptable methods of temperature measurement were those taken in situ, using a thermometer, temperature probe, or temperature data loggers.

We included temperature data reported as an average across multiple spatial replicates for each land‐use type within a study, provided that (1) the area over which data were averaged and (2) the number of spatial replicates within this area was consistent across different land‐use types within the study. We set the maximum area over which data could be averaged as 1 ha, to ensure our study focused on temperature changes at a fine spatial scale. Aggregated spatial replicates of measurements within 1 ha were considered as a single site. Where raw data were provided, a single site comprised the individual point at which measurements were taken.

We included data reported as an average across multiple temporal replicates within a study site, provided that (1) the period of time over which data were averaged and (2) the number of temporal replicates within this period was within either day or night and was consistent across different sites within the study. We set the maximum time period over which data could be averaged as 183 days (half a year), provided this time period was entirely within either the dry season or the wet season, as defined by the authors. Aggregated temporal replicates within a study site were recorded as a single observation. Where raw data provided more than one measurement per day, we calculated a daily mean for each study site (between sunrise and sunset only), each of which represented a distinct observation. If nighttime data were available, we applied the same approach for observations measured between sunset and sunrise. For those studies providing more than one temperature observation per day or night, we also calculated temperature minima and maxima for the time period(s) available (day or night).

### 2.3 Data collation

Where possible, temperature data were extracted from text, tables, or graphs in the publication. Data in graphs were extracted using DigitizeIt (www.digitizeit.de; Scheffers, Edwards, Diesmos, Williams, & Evans, 2014). We also extracted: site coordinates and elevation; site descriptions of sufficient detail to enable categorization into land‐use types; season (dry or wet); time of measurements (day or night); and whether temperature was recorded above‐ or belowground. In many cases, temperature data or methodological information was reported inadequately or not at all, in which case authors were contacted directly for information.

In some cases, we were unable to retrieve all the required methodological information and made estimates. We estimated coordinates from Google Earth, based on detailed descriptions in the text, and we estimated elevation from coordinates using a global digital elevation map at 3‐arc second resolution (NASA, SRTM NASA Version 3). Unless authors had explicitly stated that data were collected during day or night, we determined this by comparing the time of data collection to the time of sunrise and sunset, estimated from the date of collection and the site coordinates using solar calculations developed by the National Oceanic and Atmospheric Administration (NOAA Solar Calculations) and implemented in R using custom functions (https://github.com/rasenior/SolarCalc). Our main analyses use daytime temperature only because very few studies considered nighttime temperature, although we retained nighttime temperature data where they were available for an additional, simplified analysis.

We assigned categories of land use based on Extended Data Table 1 in Newbold et al. (2015), which comprise “primary forest,” “degraded forest” (renamed from “secondary”), “plantation,” “pasture,” and “cropland” (Table 1). “Urban” could not be included due to insufficient data.

### 2.4 Statistical analysis

Each data point in our main analysis comprised an observation of daytime temperature in a particular land‐use type. We modeled each temperature observation against land‐use type using a linear mixed effects model, implemented in the lme4 package (Bates, Maechler, Bolker, & Walker, 2015) in R (R Core Team 2016). Studies differed substantially in methodology and location; hence, the identity of the study from which data were taken was included as a random intercept term. Exploratory plots suggested that the slope of the relationship between land‐use type and temperature, as well as the intercept, varied by study. The decision to include a random slope of land‐use type, with respect to study identity, was determined using AIC with the full fixed effects structure (Zuur, 2009). Fixed effects were then selected using backward stepwise model simplification (Zuur, 2009), with the following categorical variables: land‐use type (five levels); position relative to ground level (above‐ or belowground); and season (dry or wet season), as well as pairwise interactions between land‐use type and the latter two variables. We tested interactions using likelihood ratio tests and then removed interactions to test main effects independently. For a subset of studies with suitable data, we used an analogous approach with only land‐use type included as a fixed effect, to model nocturnal temperature and also temperature minima and maxima (for daytime and nighttime separately).

Model estimates of local temperature are presented relative to the model estimate for primary forest (aboveground and in the dry season; Table 1). Both the position relative to ground level and seasonality interacted with land‐use change to influence local temperature, but for clarity we discuss each explanatory variable separately. As such, temperature differences between primary forest and altered land‐use types are averages across all combinations of position and season. The influence of position on these thermal differences is presented as an average across seasons, and the influence of seasonality is an average across positions.

## 3 RESULTS

**Figure 1:**
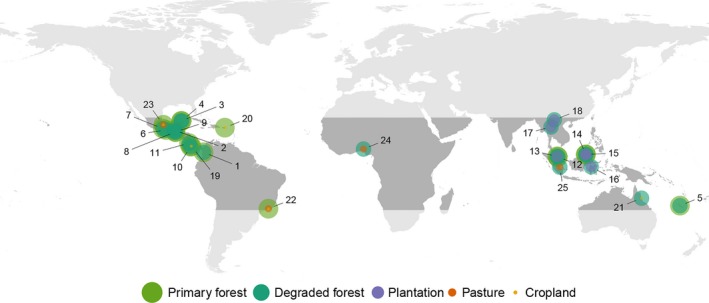
Locations of the 25 studies contributing data to the analyses. Point labels correspond to the study number in Table 1. The shading and size of concentric points corresponds to different land‐use types, to indicate the data provided by each study

**Figure 2:**
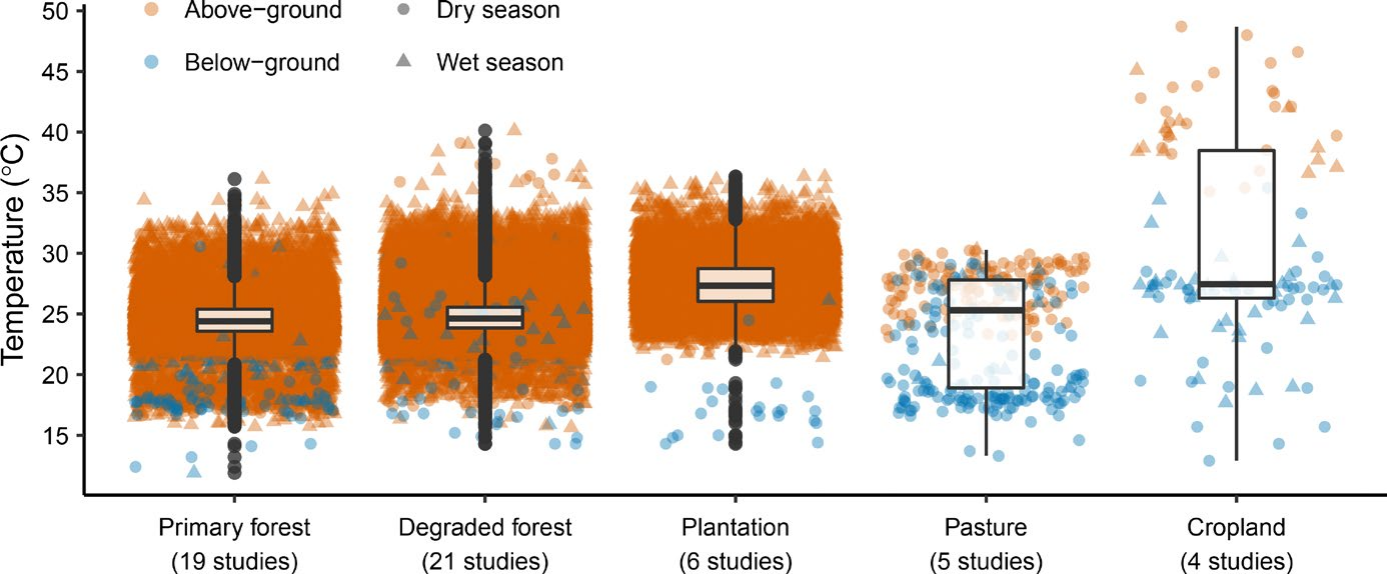
Raw daytime temperature against land‐use type, across all studies contributing data to the analyses (plotted by study in Fig. S1). Point shading indicates temperatures measured aboveground (orange) or belowground (blue), and different symbols indicate temperatures measured during the dry season (circles) or wet season (triangles)

In total, 25 studies met the criteria for inclusion (Table 2). Studies spanned 12 countries, across every continent within the tropics (Figure 1), and provided 113,894 observations of daytime temperature (Figure 2 and Fig. S1). Most observations represented either a single temperature observation within or mean temperature across, a single day at the point location where measurements were taken. Six studies reported temperature at a coarser temporal resolution (mean = 107 days; minimum = 14 days; maximum = 183 days), and six studies reported temperature at a coarser spatial resolution (mean = 527 m^2^; minimum = 64 m^2^; maximum = 1,000 m^2^). The maximum elevational difference between sites within a single study ranged from 0 to 141 m (mean = 33 m), and site elevation was random with respect to land‐use type (LMM, Χ^2^ = 19.33, *df* = 14, *p* > .05; Fig. S2). We were also able to obtain 113,459 nighttime temperature observations (including temperature extremes) from 10 studies, plus 113,230 observations of daytime temperature extremes from 11 studies; but none of these data were collected in cropland or pasture.

**Table 2:** Summary of the 25 studies contributing data to the analyses, ordered by the combination of land‐use types for which data were available. Study number corresponds to point labels in Figure 1. Crosses indicate the land‐use types, position(s) relative to ground level and season(s) considered by each study

Study	Country	Land‐use type					Position		Season	
		Primary forest	Degraded forest	Plantation	Pasture	Cropland	Aboveground	Belowground	Dry season	Wet season
1. González del Pliego (Unpublished data)	Colombia	X	X				X		X	
2. González‐Di Pierro et al. (2011)	Mexico	X	X				X			X
3. Goode (Unpublished data)	Mexico	X	X				X		X	X
4. Goode and Allen (2009)	Mexico	X	X				X		X	X
5. Ibanez, Hély, and Gaucherel (2013)	New Caledonia	X	X				X		X	X
6. Lebrija‐Trejos, Pérez‐García, Meave, Poorter, and Bongers (2011)	Mexico	X	X				X	X	X	X
7. Negrete‐Yankelevich, Fragoso, Newton, and Heal (2007)	Mexico	X	X					X		X
8. Santos (2011)	Mexico	X	X				X	X		X
9. Santos and Benítez‐Malvido (2012)	Mexico	X	X				X	X		X
10. Sonnleitner, Dullinger, Wanek, and Zechmeister (2009)	Costa Rica	X	X				X		X	
11. Wood and Lawrence (2008)	Costa Rica	X	X					X		X
12. Yashiro, Kadir, Okuda, and Koizumi (2008)	Malaysia	X	X					X	X	X
13. Adachi, Bekku, Rashidah, Okuda, and Koizumi (2006)	Malaysia	X	X	X				X	X	
14. Hardwick and Orme (2016)	Malaysia	X	X	X			X		X	X
15. Hardwick et al. (2015)	Malaysia	X	X	X			X		X	X
16. Klein, Steffan‐Dewenter, and Tscharntke (2002)	Indonesia		X	X			X			X
17. Wangluk, Boonyawat, Diloksumpun, and Tongdeenok (2013)	Thailand		X	X				X	X	X
18. Werner et al. (2006)	China		X	X				X	X	
19. Holl (1999)	Costa Rica	X			X		X	X	X	
20. Liu and Zou (2002)	Puerto Rico	X			X			X	X	X
21. King, Andersen, and Cutter (1998)	Australia		X		X		X	X		X
22. Badejo, De Aquino, De‐Polli, and Correia (2004)	Brazil	X			X	X		X		X
23. Campos (2006)	Mexico	X			X	X		X	X	X
24. Badejo (1990)	Nigeria		X			X		X	X	X
25. Furukawa, Inubushi, Ali, Itang, and Tsuruta (2005)	Indonesia		X			X	X	X	X	X

In all cases, the final model included a random slope for land‐use type (“LUT”) and random intercept with respect to the identity of the study (“studyID”) from which data originated. The final model of daytime temperature (“temp_day_”) included land‐use type, position relative to ground level (“position”) and season, as well as pairwise interactions between land‐use type and the latter two fixed effects:

$$\text{lmer}\left(\text{temp}{}_{\mathrm{day}}∼\text{LUT}*\text{position}+\text{LUT}*\text{season}+\left(\text{LUT}|\text{studyID}\right)\right)$$

The final models of (1) nighttime temperature, and temperature extremes (minimum and maximum) (2) during the day and (3) during the night, all had the same model structure, with land‐use type as the only fixed effect:

$$\text{lmer}\left(\text{temp}∼\text{LUT}+\left(\text{LUT}|\text{studyID}\right)\right)$$

### 3.1 Effect of land‐use change

**Figure 3:**
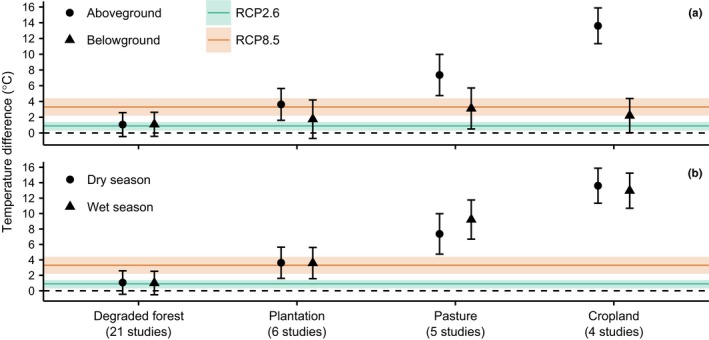
Model estimates of local daytime temperature in altered land‐use types relative to primary forest (depicted by the black dashed line). In Panel A, different symbols denote position relative to the ground (above‐ or belowground), and the season is held at the reference level (dry season). In Panel B, different symbols denote the season (dry or wet), and the position relative to the ground is held at the reference level (aboveground). Error bars are 95% confidence intervals. Solid lines indicate projected warming in the tropics for the period 2081–2100 compared to the period 1986–2005, as a result of global climate change (IPCC, 2013). Shaded bands indicate 5%–95% ranges from the distribution of the climate model ensemble. Colors represent the lowest and highest warming scenarios (RCP2.6 and RCP8.5, respectively)

Altered land‐use types were substantially hotter than primary forest (LMM, Χ^2^ = 29.49, *df* = 4, *p* *<* .001; Table 3; Figure 3), and the magnitude of the warming broadly matched the intensity of vegetation change associated with each land‐use type. Thus, degraded forests in our sample were the most similar to primary forest with an average difference of only +1.1°C, which was not statistically significant based on 95% confidence intervals (Figure 3). By contrast, converted habitats in our dataset—plantation, pasture, and cropland—were, on average, hotter than primary forest by 2.7°C, 6.2°C, and 7.6°C, respectively. Results were robust to resampling from studies that provided disproportionate numbers of observations (Supporting Information Text S1 and Fig. S3).

**Table 3:** Model estimates (with 95% confidence intervals) of local daytime temperature in altered land‐use types relative to primary forest (PF), with respect to position relative to ground level and season. ‘Position effect’ refers to the difference between temperature measured aboveground (AG) versus belowground (BG), averaged across seasons. ‘Season effect’ refers to the difference between temperature measured in the dry season versus the wet season, averaged across positions. All figures are quoted in °C

Land‐use type (LUT)	Position	Season	Temp. versus PF	Lower CI	Upper CI	LUT mean	Position	Position mean	Position effect (AG–BG)	Season	Season mean	Season effect (dry–wet)
Degraded forest	Aboveground	Dry	1.1	−0.5	2.6	1.1	Aboveground	1	0.1	Dry	1.1	0.1
		Wet	1	−0.5	2.5					Wet	1	
	Belowground	Dry	1.1	−0.4	2.6		Belowground	1.1				
		Wet	1	−0.5	2.6							
Plantation	Aboveground	Dry	3.6	1.6	5.6	2.7	Aboveground	3.6	1.9	Dry	2.7	0.1
		Wet	3.6	1.6	5.6					Wet	2.6	
	Belowground	Dry	1.8	−0.7	4.2		Belowground	1.7				
		Wet	1.7	−0.7	4.2							
Pasture	Aboveground	Dry	7.4	4.7	10	6.2	Aboveground	8.3	4.3	Dry	5.2	−1.9
		Wet	9.2	6.7	11.8					Wet	7.1	
	Belowground	Dry	3.1	0.5	5.7		Belowground	4				
		Wet	5	2.4	7.5							
Cropland	Aboveground	Dry	13.6	11.3	15.9	7.6	Aboveground	13.3	11.4	Dry	7.9	0.6
		Wet	13	10.7	15.2					Wet	7.3	
	Belowground	Dry	2.2	0	4.4		Belowground	1.9				
		Wet	1.6	−0.6	3.7							

Nighttime temperature, and daytime and nighttime temperature extremes, showed varying results relative to primary forest in the two altered land‐use types for which data were available: degraded forest and plantation. In all cases, sample sizes were very limited and confidence intervals were large; hence, results should be interpreted with caution. Nighttime temperature in degraded forest and plantation did not differ from that of primary forest (LMM, Χ^2^ = 2.09, *df* = 2, *p* > .05; Fig. S4), and neither did nighttime minimum temperature (LMM, Χ^2^ = 2.31, *df* = 2, *p* > .05; Fig. S5D). Maximum nighttime temperature was slightly higher overall in degraded forest and plantation compared to primary forest (LMM, Χ^2^ = 6.35, *df* = 2, *p* < .05; Fig. S5C), although pairwise differences were not statistically significant according to 95% confidence intervals. There was no difference between primary forest and degraded forest and plantation in terms of daytime maximum temperature (LMM, Χ^2^ = 4.87, *df* = 2, *p* > .05; Fig. S5A), or daytime minimum temperature (LMM, Χ^2^ = 4.60, *df* = 2, *p* > .05; Fig. S5B).

### 3.2 Above‐ versus belowground

The warming effect of land‐use change was much stronger aboveground than belowground (LMM, Χ^2^ = 1115, *df* = 4, *p* *<* .001; Table 3; Figure 3a). The average difference between the local temperature of altered land‐use types and primary forest was greater if measured aboveground rather than belowground, by 1.9°C in plantation, 4.3°C in pasture, and 11.4°C in cropland. In degraded forest, the temperature relative to primary forest was very similar above‐ (+1°C) and belowground (+1.1°C). Notably, the buffering effect below ground was so great that any difference between primary forest and impacted land uses was effectively negated in all land‐use types but pasture (based on 95% confidence intervals; Figure 3a).

### 3.3 Dry versus wet season

Seasonality had some influence on the relationship between land‐use change and temperature (LMM, Χ^2^ = 14.91, *df* = 4, *p* < .01; Table 3; Figure 3b), but the direction of the interaction varied by land‐use type, and in all cases the effect size was very small. In degraded forest and plantation, seasonality had no appreciable effect on temperature relative to primary forest (dry vs. wet season: +0.1°C in both degraded forest and plantation). In contrast, the temperature difference between pasture and primary forest was 1.9°C greater in the wet versus dry season; while in cropland, the differential was 0.6°C greater in the dry versus wet season.

## 4 DISCUSSION

Our results show that land‐use change increases local temperature in the tropics (Figure 3). In all conditions where this relationship was evident, the temperature rise due to land‐use change exceeded that predicted for the tropics by the end of the 21st Century under the minimum climate warming scenario (+0.9°C in RCP2.6; IPCC 2013), and frequently also exceeded the maximum warming scenario (+3.3°C in RCP8.5; IPCC 2013). Previous studies show that land‐use change tends to increase local temperature (e.g., Davin & De Noblet‐Ducoudr, 2010; Findell et al., 2007; Loarie et al., 2009; Luskin & Potts, 2011; Ramdani et al., 2014; Tuff et al., 2016) but this is the first study, to our knowledge, that demonstrates this effect across many locations in the tropics at a site‐level resolution (<1 ha), considering multiple modes of land‐use change concurrently, and comparing the relationship above‐ and belowground and between wet and dry seasons.

### 4.1 Thermal differences between land‐use types

Human‐impacted land‐use types are likely hotter than intact primary forest because of changes in evapotranspiration and the amount of solar radiation reaching the Earth's surface (Davin & De Noblet‐Ducoudr, 2010; Findell et al., 2007; Oke, 1987). Degradation and deforestation cause a lowering and thinning of the canopy, and reduction in rooting depth, leaf area index, and surface roughness, all of which reduce evapotranspiration (Davin & De Noblet‐Ducoudr, 2010; Findell et al., 2007; Hardwick et al., 2015; Kumar & Shahabuddin, 2005; Okuda et al., 2003; Snyder et al., 2004), and thereby increase temperature (Foley et al., 2005; Oke, 1987). Changes to canopy architecture and a reduction in the number of subcanopy vegetation strata also cause warming by increasing the amount of solar radiation reaching the ground (Murcia, 1995; Oke, 1987). Our land‐use categories encompass a spectrum of vegetation change, from relatively little change in degraded forests (where some trees and a closed canopy are maintained) to maximal change in pasture and cropland (where trees are replaced with herbaceous plants). Accordingly, degradation had the smallest average effect (+1.1°C), followed by plantation (+2.7°C), and then pasture (+6.2°C) and cropland (+7.6°C).

We expected that the same mechanisms underlying the warming effect of land‐use change would also result in increased daytime temperature extremes and decreased nighttime temperatures in altered land‐use types, relative to primary forest (Chen et al., 1995; Oke, 1987)**.** Unfortunately, the data available were very limited, including only three of the five land‐use types (primary forest, degraded forest and plantation), and resulting in extremely large confidence intervals (Figs. S3 and S4). We urge caution when interpreting our results, which suggested either no effect or an extremely weak effect of land‐use change on temperature extremes and nighttime temperature; clearly more data are needed to reliably test these relationships.

### 4.2 Interaction with position relative to ground level and seasonality

We found that local warming effects of tropical land‐use change are negated belowground, despite the strength of the relationship aboveground (Table 3; Figure 3a). This can largely be attributed to the higher specific heat capacity of soil compared to air (Oke, 1987). Greater availability of water may also play a role, permitting thermal energy to be dissipated through the evaporation of water rather than increasing temperature (Christidis et al., 2013; Davin & De Noblet‐Ducoudr, 2010; Oke, 1987). We expected the latter effect to result in increased buffering during the wet season (cf. Davin & De Noblet‐Ducoudr, 2010; Findell et al., 2007), but instead we found that seasonality had a very limited influence on temperature relative to primary forest (Table 3; Figure 3b). The strongest influence was in pasture, where the effect of land‐use change was greater in the wet season. Potentially longer grass in pasture in the wet season could decrease albedo compared to pale exposed soil in the dry season, while the same pattern could be avoided in cropland through dry season irrigation. That said, pasture and cropland had the least data of all land‐use types, and we advise that these results be interpreted with caution.

### 4.3 Implications for biodiversity

For tropical biodiversity, there are several key implications of our findings. Firstly, forest species persisting through forest conversion have already experienced thermal change similar, if not greater, in magnitude to that predicted by global climate change (IPCC 2013). Historically the tropics have experienced relatively stable climatic conditions (Mora et al., 2013) and tropical species possess narrow thermal niches, with many already occupying the upper bounds of that niche (Deutsch et al., 2008; Freeman & Freeman, 2014; Sunday et al., 2014; Tewksbury et al., 2008). Dispersal toward more favorable climatic conditions is limited by low dispersal ability (Van Houtan et al., 2007), a scarcity of suitable destinations (Colwell et al., 2008), and the necessity to pass through an increasingly hostile land‐use matrix to reach target habitat (Brook et al., 2008; Scriven et al., 2015; Thomas et al., 2004). There is already some evidence that higher temperatures in the tropics are associated with lower species abundance (e.g., for arthropods: Foster et al., 2011), and there are also fitness costs associated with long‐term persistence in suboptimal climatic conditions (Du Plessis, Martin, Hockey, Cunningham, & Ridley, 2012; Gunderson & Leal, 2016). Without any further temperature change, some species persisting in converted environments may already be committed to extinction, particularly species that are unable to utilize microhabitats with favorable microclimates (González Del Pliego et al., 2016; Scheffers, Evans, et al., 2014). Under predicted climate change, increasing average temperature and the increasing frequency and intensity of droughts (Chou & Lan, 2012; IPCC 2013) will likely push many species beyond their upper thermal limits, especially in heavily degraded or converted habitats.

That said, we find several circumstances where warming through land‐use change is mitigated. Degraded forests were not significantly hotter than primary forests (according to 95% confidence intervals; Figure 3). This is encouraging because degraded forests are likely to become the most widespread land‐use type in the future (Hurtt et al., 2011), and many studies have demonstrated their capacity to retain species of conservation concern (Edwards, Tobias, Sheil, Meijaard, & Laurance, 2014; Edwards et al., 2011; Gibson et al., 2011; Putz et al., 2012). For all altered land‐use types, the warming effect was limited belowground, highlighting a crucial thermal refuge for species that are able to occupy the soil, and suggesting that aboveground microhabitats, such as deadwood and epiphytes, might fulfill a similar role (González Del Pliego et al., 2016; Scheffers, Edwards, et al., 2014; Scheffers, Evans, et al., 2014). Thermal refugia may not be a permanent solution for avoiding climate change, and sensitive species may find that even relatively cold microhabitats are still too hot (e.g., belowground in pasture was 4°C warmer than primary forest; Table 3; Figure 3), but refugia could at least provide species with more time to respond to suboptimal climatic conditions (Hannah et al., 2014).

### 4.4 Caveats and knowledge gaps

By collating site‐level data reported from the literature, we were able to achieve high geographical coverage and fine spatial resolution that is lacking in previous studies, but this technique is biased by the availability of data toward particular regions and land‐use types (Figure 1) and relies heavily on substituting space for time, which can misrepresent anthropogenic impacts (França et al., 2016). In particular, there was only one study located in Africa, and Southeast Asian studies provided all of the plantation data and no cropland data. Future research should seek to explicitly consider how tropical land‐use change affects: vegetation structure (e.g., using Leaf Area Index cf. Hardwick et al., 2015), relative humidity (Ewers & Banks‐Leite, 2013; Luskin & Potts, 2011), nocturnal climatic conditions (Chen et al., 1995; Dubreuil, Debortoli, Funatsu, Nédélec, & Durieux, 2011), extremes of temperature (Christidis et al., 2013), and rates of temperature change (Scheffers, Evans, et al., 2014); preferably at a range of spatiotemporal scales (Wiens & Bachelet, 2010) and with a standardized methodology to simplify comparisons across studies.

## 5 CONCLUSIONS

Our study confirms that tropical land‐use change leads to warming at a local scale (<1 ha) across the tropics, of a magnitude comparable to that predicted from global climate change. We find pantropical evidence that the effects of land‐use change on temperature are ameliorated belowground, and absent in degraded forests. Many studies collect site‐level climate data, and through sharing of these data and collaboration between scientific disciplines, there is much that can be carried out to integrate theoretical and empirical understanding of the processes that govern climate at different scales. This will greatly advance our knowledge of potential synergies between two of the greatest drivers of biodiversity loss—land‐use change and climate change—and highlight mitigating factors, such as thermal microrefugia, which could be a pragmatic focus for conservation management.

## DATA AND R CODE

The collated dataset can be found on Dryad (https://doi.org/10.5061/dryad.g4000). Note that in many cases, these data were aggregated for analyses. For finer resolution data, please refer to the original data sources. R functions used to estimate time of sunset and sunrise can be downloaded from GitHub (https://github.com/rasenior/SolarCalc).

## CONFLICT OF INTEREST

Authors declare no conflicts of interest.

## AUTHOR CONTRIBUTIONS

R.A.S., D.P.E., and J.K.H conceived the study. R.A.S., P.G. and L.K.G. collated the data. R.A.S. performed statistical analyses. R.A.S. wrote the manuscript, with substantial editorial contributions from D.P.E. and J.K.H.
