# Supplementary material for: A pantropical analysis of the impacts of forest degradation and conversion on local temperature
Source: Ecol Evol. 2017 Aug 30;7(19):7897–908. doi: 10.1002/ece3.3262 (PMC5632667; doi:10.1002/ece3.3262)
Supplement: Supplementary file 1 [file ECE3-7-7897-s001.pdf]

A pan-tropical analysis of the impacts of forest degradation and conversion on local temperature

Rebecca A. Senior[1*], Jane K. Hill[2], Pamela González del Pliego[1], Laurel K. Goode[3] and David P. Edwards[1]

[1]Department of Animal and Plant Sciences, Alfred Denny Building, University of Sheffield, Western Bank, Sheffield, S10 2TN, UK

[2]Department of Biology, University of York, Wentworth Way, York, YO10 5DD, UK

[3]Department of Human Services and Oregon Health Authority, Salem, Oregon, USA

[*]**Corresponding author:** rebecca.a.senior@gmail.com (R.A. Senior)

Text S1: Impact of unbalanced sampling

Methods

Some studies contributed substantially more temperature observations than others. To test whether these studies were unduly influencing our results, we established a threshold over which a given land-use type, in a given study, was deemed to have a disproportionate number of associated temperature observations. The threshold used – 2,071 observations – was the mean number of observations across all unique combinations of land-use type and study identity (55 in total). The same number of observations (2,071) was then randomly re-sampled from each of the land-use type and study combinations that exceeded the threshold. With this reduced and more balanced dataset we repeated the main analysis (see 'Statistical analysis' in main text for more details), modelling local day-time temperature ('$temp_{day}$') against land-use type ('LUT'), position relative to ground-level ('position') and season. The final model structure was unchanged, and included a random slope for land-use type and random intercept with respect to the identity of the study ('studyID') from which data originated:

$$lmer(temp_{day} \sim LUT*position + LUT*season + (LUT|studyID))$$

Results

All results were qualitatively unchanged from those derived using the full dataset. Local day-time temperature was warmer in altered land-use types, compared to primary forest (LMM, $X^2 = 32.19$, df = 4, $P < 0.001$; Fig. S3). Averaged across model estimates for above- and below-ground, and across seasons, the temperature differential was greatest in cropland (7.7°C), followed by pasture (6.4°C), plantation (3.2°C) and degraded forest (0.9°C).

The relationship between land-use type and temperature interacted with both position relative to ground level (LMM, $X^2 = 681$, df = 4, $P < 0.001$; Fig. S3A) and season (LMM, $X^2 = 105.63$, df = 4, P <

0.001; Fig. S3B). Specifically, the difference between altered land-use types and primary forest was greater above-ground than below-ground (Fig. S3A), and variable between seasons according to the land-use type (Fig. S3B).

Supplementary figures

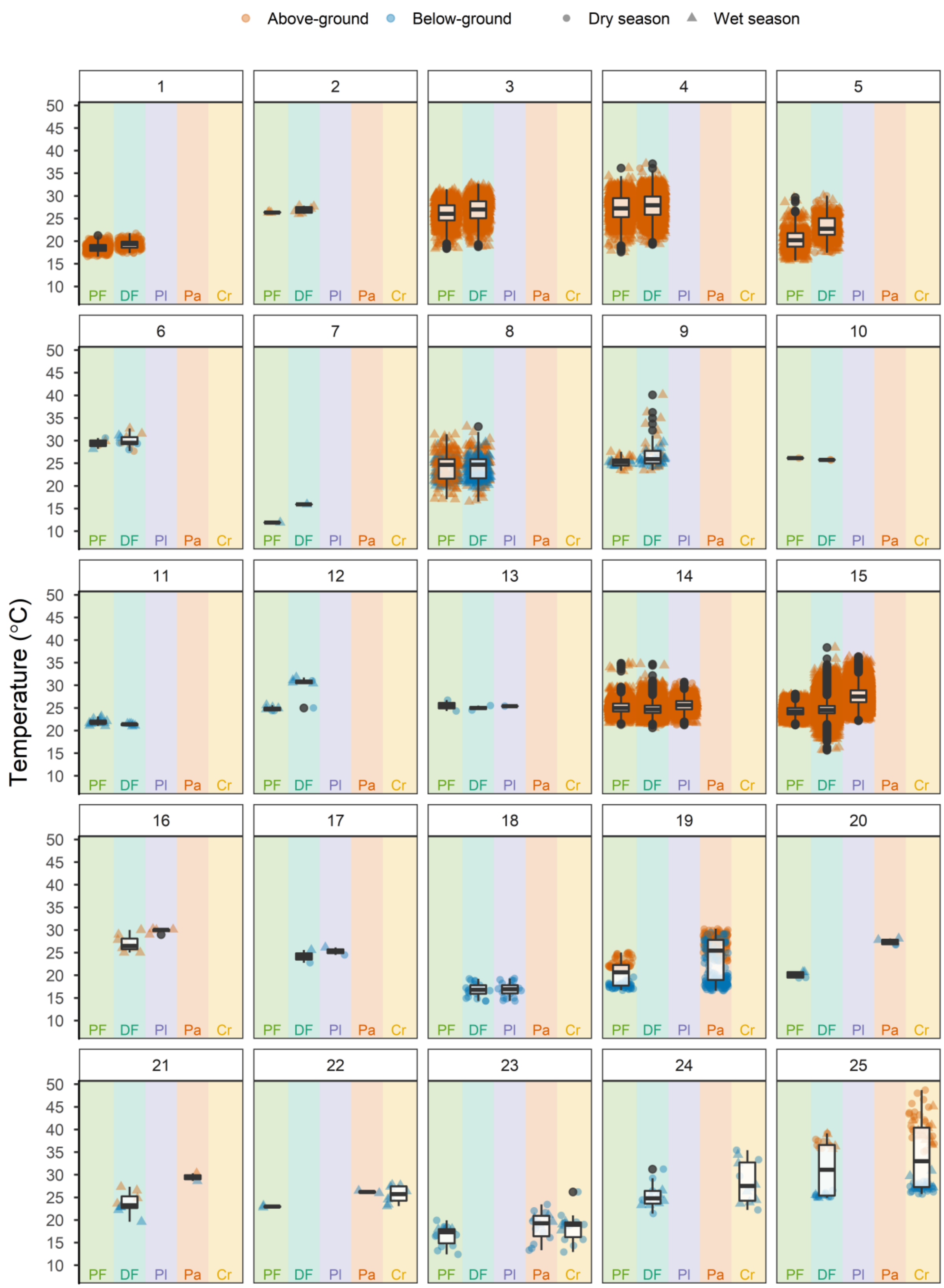

Figure S1. Raw day-time temperature against land-use type for each study contributing data to the analyses. Panel numbers refer to the study number in the reference list below. Land-use types are:

primary forest (PF), degraded forest (DF), plantation (Pl), pasture (Pa) and cropland (Cr). Panels are ordered by the combination of land-use types for which data was available: (1-12) PF + DF; (13-15) PF + DF + Pl; (16-18) DF + Pl; (19-20) PF + Pa; (21) DF + Pa; (22-23) PF + Pa + Cr; and (24-25) DF + Cr. Shading of points indicates temperatures measured above-ground (orange) or below-ground (blue), and point symbol indicates temperatures measured during the dry season (circles) or wet season (triangles).

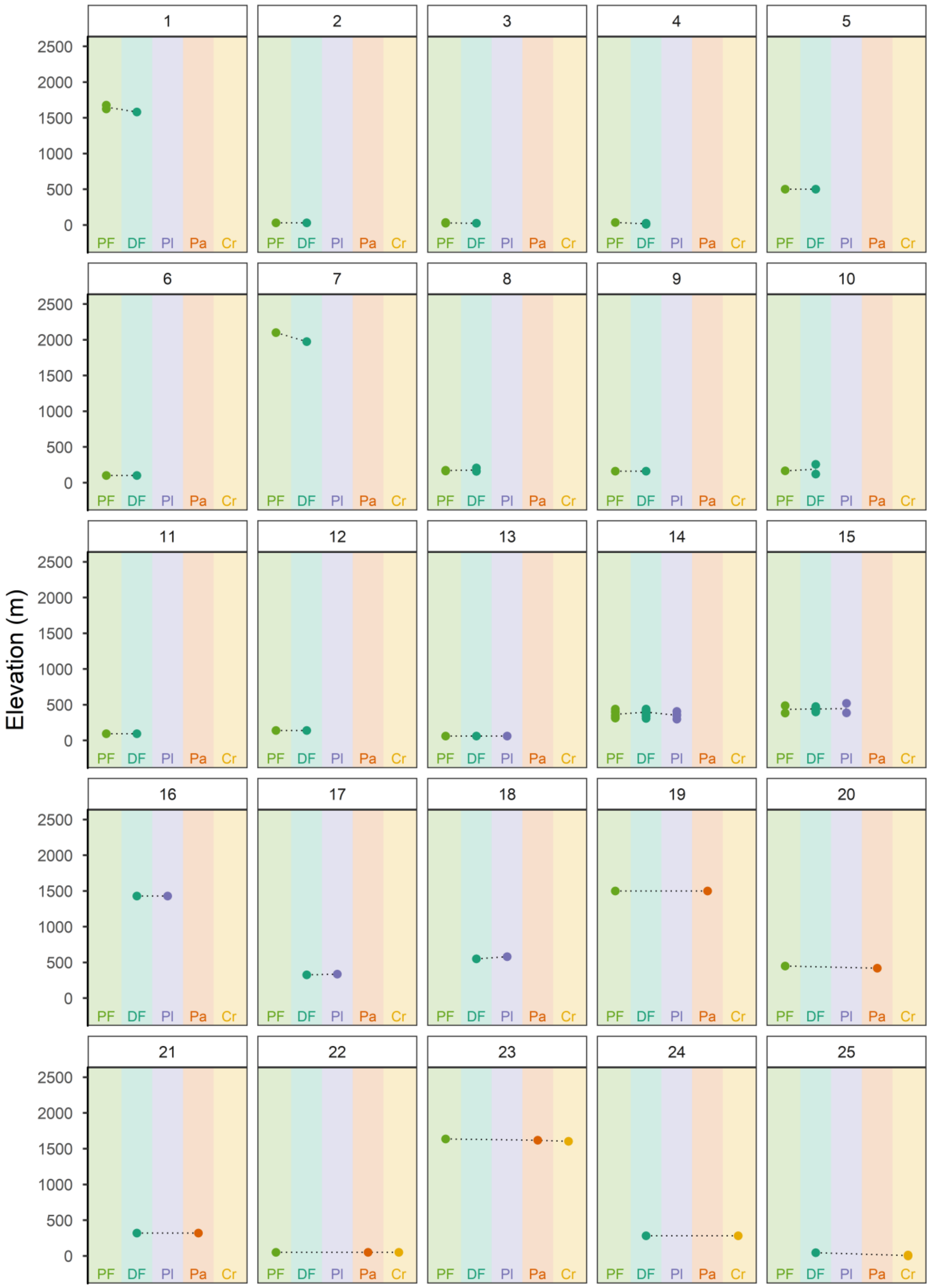

Figure S2. Site elevation against land-use type for each study contributing data to the analyses. Panel numbers refer to the study number in the reference list below. Land-use types are: primary forest (PF), degraded forest (DF), plantation (Pl), pasture (Pa) and cropland (Cr). Panels are ordered by the

combination of land-use types for which data was available: (1-12) PF + DF; (13-15) PF + DF + Pl; (16-18) DF + Pl; (19-20) PF + Pa; (21) DF + Pa; (22-23) PF + Pa + Cr; and (24-25) DF + Cr. Dotted black lines connect the mean elevation of all the sites within each land-use type.

Reference list for the study numbers denoted in the panels of Figures S1 and S2.

1. González del Pliego P (Unpublished data)

2. González-Di Pierro AM, Benítez-Malvido J, Méndez-Toribio M, et al (2011) Effects of the physical environment and primate gut passage on the early establishment of *Ampelocera hottlei Standley* in rain forest fragments. Biotropica 43:459–466. doi: 10.1111/j.1744-7429.2010.00734.x

3. Goode LK (Unpublished data)

4. Goode LK, Allen MF (2009) Seed germination conditions and implications for establishment of an epiphyte, *Aechmea bracteata* (Bromeliaceae). Plant Ecol 204:179–188. doi: 10.1007/s11258-009-9582-7

5. Ibanez T, Hély C, Gaucherel C (2013) Sharp transitions in microclimatic conditions between savanna and forest in New Caledonia: Insights into the vulnerability of forest edges to fire. Austral Ecol 38:680–687. doi: 10.1111/aec.12015

6. Lebrija-Trejos E, Pérez-García EA, Meave JA, et al (2011) Environmental changes during secondary succession in a tropical dry forest in Mexico. J Trop Ecol 27:477–489. doi: 10.1017/S0266467411000253

7. Negrete-Yankelevich S, Fragoso C, Newton AC, Heal OW (2007) Successional changes in soil, litter and macroinvertebrate parameters following selective logging in a Mexican Cloud Forest. Appl Soil Ecol 35:340–355. doi: 10.1016/j.apsoil.2006.07.006

8. Santos BA (2011) La interacción de heliconia con sus insectos herbívoros y hongos patógenos foliares en selvas tropicales fragmentadas. PhD thesis, Centro de Investigaciones en Ecosistemas, Universidad Nacional Autónoma de México, Mexico.

9. Santos BA, Benítez-Malvido J (2012) Insect herbivory and leaf disease in natural and human disturbed habitats: lessons from early-successional *Heliconia* herbs. Biotropica 44:53–62. doi: 10.1111/j.1744-7429.2011.00765.x

10. Sonnleitner M, Dullinger S, Wanek W, Zechmeister H (2009) Microclimatic patterns correlate with the distribution of epiphyllous bryophytes in a tropical lowland rain forest in Costa Rica. J Trop Ecol 25:321–330. doi: 10.1017/S0266467409006002

11. Wood TE, Lawrence D (2008) No short-term change in soil properties following four-fold litter addition in a Costa Rican rain forest. Plant Soil 307:113–122. doi: 10.1007/s11104-008-9588-2

12. Yashiro Y, Kadir WR, Okuda T, Koizumi H (2008) The effects of logging on soil greenhouse gas (CO_2, CH_4, N_2O) flux in a tropical rain forest, Peninsular Malaysia. Agric For Meteorol 148:799–806. doi: 10.1016/j.agrformet.2008.01.010

13. Adachi M, Bekku YS, Rashidah W, et al (2006) Differences in soil respiration between different tropical ecosystems. Appl Soil Ecol 34:258–265. doi: 10.1016/j.apsoil.2006.01.006

14. Hardwick S, Orme D (2016) Aboveground microclimate at SAFE 2013 - 2015. Zenodo. doi: 10.5281/zenodo.46183

15. Hardwick SR, Toumi R, Pfeifer M, et al (2015) The relationship between leaf area index and microclimate in tropical forest and oil palm plantation: Forest disturbance drives changes in microclimate. Agric For Meteorol 201:187–195. doi: 10.1016/j.agrformet.2014.11.010

16. Klein A-M, Steffan-Dewenter I, Tscharntke T (2002) Predator–prey ratios on cocoa along a land-use gradient in Indonesia. Biodivers Conserv 11:683–693. doi: 10.1023/A:1015548426672

17. Wangluk S, Boonyawat S, Diloksumpun S, Tongdeenok P (2013) Role of soil temperature and moisture on soil respiration in a teak plantation and mixed deciduous forest in Thailand. J Trop For Sci 339–349.

18. Werner C, Zheng X, Tang J, et al (2006) N_2O, CH_4 and CO_2 emissions from seasonal tropical rainforests and a rubber plantation in Southwest China. Plant Soil 289:335–353. doi: 10.1007/s11104-006-9143-y

19. Holl KD (1999) Factors limiting tropical rain forest regeneration in abandoned pasture: seed rain, seed germination, microclimate, and soil. Biotropica 31:229–242.

20. Liu ZG, Zou XM (2002) Exotic earthworms accelerate plant litter decomposition in a Puerto Rican pasture and a wet forest. Ecol Appl 12:1406–1417. doi: 10.1890/1051-0761(2002)012[1406:EEAPLD]2.0.CO;2

21. King JR, Andersen AN, Cutter AD (1998) Ants as bioindicators of habitat disturbance: validation of the functional group model for Australia's humid tropics. Biodivers Conserv 7:1627–1638. doi: 10.1023/A:1008857214743

22. Badejo MA (1990) Seasonal abundance of soil mites (Acarina) in two contrasting environments. Biotropica 22:382–390. doi: 10.2307/2388555

23. Campos CA (2006) Response of soil surface CO_2-C flux to land use changes in a tropical cloud forest (Mexico). For Ecol Manag 234:305–312. doi: 10.1016/j.foreco.2006.07.012

24. Badejo MA, De Aquino AM, De-Polli H, Correia MEF (2004) Response of soil mites to organic cultivation in an ultisol in southeast Brazil. Exp Appl Acarol 34:345–364.

25. Furukawa Y, Inubushi K, Ali M, et al (2005) Effect of changing groundwater levels caused by land-use changes on greenhouse gas fluxes from tropical peat lands. Nutr Cycl Agroecosystems 71:81–91. doi: 10.1007/s10705-004-5286-5

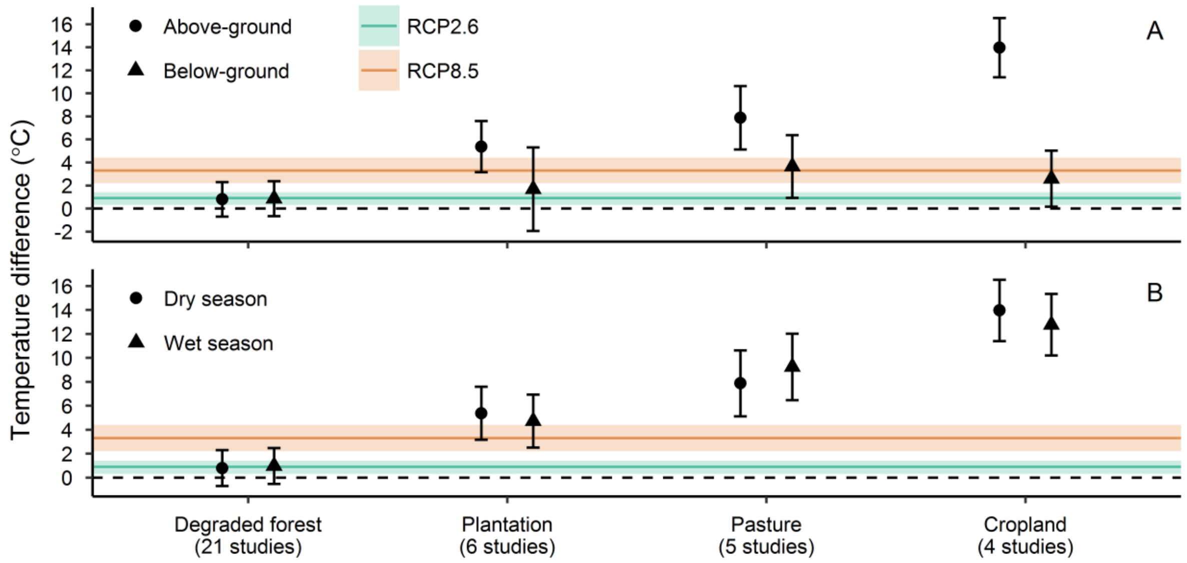

Figure S3. Model estimates of local day-time temperature in altered land-use types relative to primary forest (depicted by the black dashed line) using a reduced dataset to balance sample sizes between the different studies that contributed data. Error bars are 95% confidence intervals. Solid lines indicate projected warming in the tropics for the period 2081-2100 compared to the period 1986-2005, as a result of global climate change (IPCC, 2013). Shaded bands indicate 5 to 95% ranges from the distribution of the climate model ensemble. Colours represent the lowest and highest warming scenarios (RCP2.6 and RCP8.5, respectively).

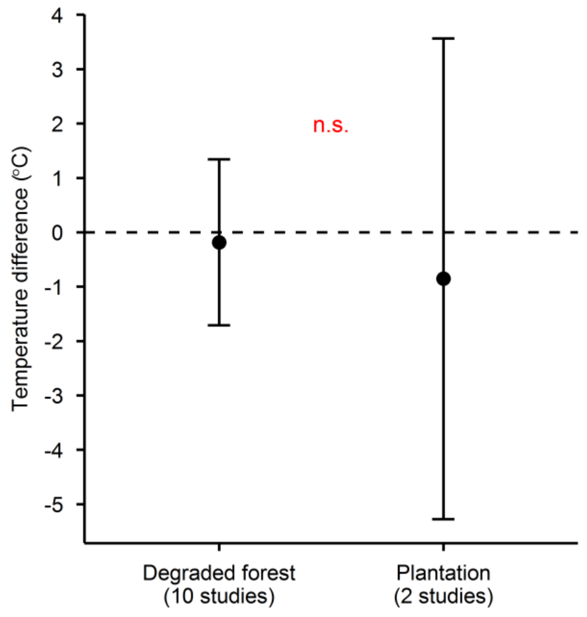

Figure S4. Model estimates of local night-time temperature in altered land-use types relative to primary forest (depicted by the black dashed line). Note that cropland and pasture are missing from this analysis because nocturnal temperature data for these land-use types were not available. Error bars are 95% confidence intervals.

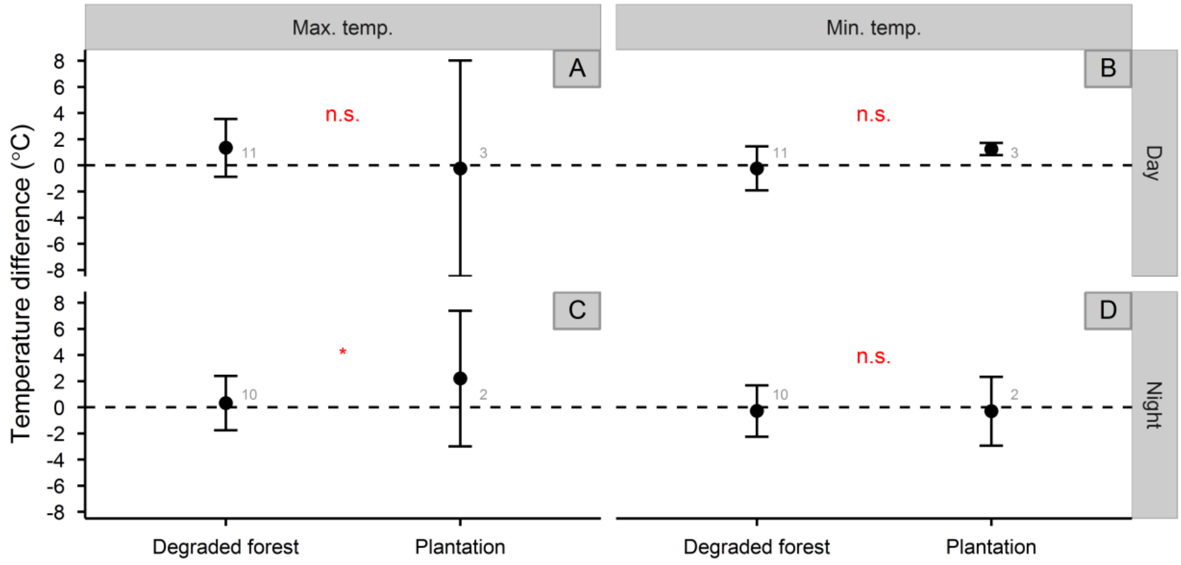

Figure S5. Model estimates of local temperature extremes in altered land-use types relative to primary forest (depicted by the black dashed line). Day-time results are depicted in panels A and B, and night-time results in panels C and D. Panels A and C indicate the effect of land-use change on maximum temperature, and panels B and D indicate the same for minimum temperature. Note that data for cropland and pasture are absent from this analysis because data for these land-use types were not available. Error bars are 95% confidence intervals. The grey numbers next to points represent the number of studies providing the underlying data.
